# Performance Diagnóstica da FFR por Angiotomografia de Coronárias através de Software Baseado em Inteligência Artificial

**DOI:** 10.36660/abc.20190329

**Published:** 2021-06-08

**Authors:** Thamara Carvalho Morais, Antonildes Nascimento Assunção-Jr, Roberto Nery Dantas, Carla Franco Grego da Silva, Caroline Bastida de Paula, Roberto Almeida Torres, Tiago Augusto Magalhães, César Higa Nomura, Luiz Francisco Rodrigues de Ávila, José Rodrigues Parga

**Affiliations:** 1 Hospital Sírio-libanês São PauloSP Brasil Hospital Sírio-libanês , São Paulo , SP - Brasil; 2 Universidade de São Paulo Faculdade de Medicina Departamento de Imagem Cardiovascular São PauloSP Brasil Universidade de São Paulo Faculdade de Medicina - CDI - InCor/HCFMUSP - Departamento de Imagem Cardiovascular , São Paulo , SP - Brasil; 3 Complexo Hospital de Clínicas Universidade Federal do Paraná CuritibaPR Brasil Complexo Hospital de Clínicas da Universidade Federal do Paraná (CHC-UFPR) - Cardiovascular CT/MR, Curitiba , PR - Brasil

**Keywords:** Reserva Fracionada de Fluxo Miocárdico, Doença Arterial Coronariana, Tomografia Computadorizada, Isquemia Miocárdica, Aprendizado de Máquina

## Abstract

**Fundamento:**

A quantificação não invasiva da reserva fracionada de fluxo miocárdico (FFR _TC_ ) através de *software* baseado em inteligência artificial em versão mais atualizada e tomógrafo de última geração (384 cortes) apresenta elevada *performance* na detecção de isquemia coronariana.

**Objetivos:**

Avaliar o desempenho diagnóstico da FFR _TC_ na detecção de doença arterial coronariana (DAC) significativa em relação ao FFRi, em tomógrafos de gerações anteriores (128 e 256 cortes).

**Métodos:**

Estudo retrospectivo com pacientes encaminhados à angiotomografia de artérias coronárias (TCC) e cateterismo (FFRi). Foram utilizados os tomógrafos Siemens Somatom Definition Flash (256 cortes) e AS+ (128 cortes). A FFR _TC_ e a área luminal mínima (ALM) foram avaliadas em *software* (cFFR versão 3.0.0, Siemens Healthineers, Forchheim, Alemanha). DAC obstrutiva foi definida como TCC com redução luminal ≥50% e DAC funcionalmente obstrutiva como FFRi ≤0,8. Todos os valores de *p* reportados são bicaudais; e quando <0,05, foram considerados estatisticamente significativos.

**Resultados:**

Noventa e três pacientes consecutivos (152 vasos) foram incluídos. Houve boa concordância entre FFR _TC_ e FFRi, com mínima superestimação da FFR _TC_ (viés: –0,02; limites de concordância: 0,14 a 0,09). Diferentes tomógrafos não modificaram a relação entre FFR _TC_ e FFRi (p para interação = 0,73). A FFR _TC_ demonstrou *performance* significativamente superior à classificação visual de estenose coronariana (AUC 0,93 *vs.* 0,61, p <0,001) e à ALM (AUC 0,93 *vs.* 0,75, p <0,001) reduzindo o número de casos falso-positivos. O melhor ponto de corte para a FFR _TC_ utilizando um índice de Youden foi de 0,85 (sensiblidade, 87%; especificidade, 86%; VPP, 73%; NPV, 94%), com redução de falso-positivos.

**Conclusão:**

FFR _TC_ baseada em inteligência artificial, em tomógrafos de gerações anteriores (128 e 256 cortes), apresenta boa *performance* diagnóstica na detecção de DAC, podendo ser utilizada para reduzir procedimentos invasivos.

## Introdução

De acordo com as diretrizes clínicas mais recentes, ^1 - 3^ o manejo da doença arterial coronariana (DAC) crônica e sintomática pode ser guiado por testes adicionais para avaliação anatômica (extensão, severidade, morfologia) ou funcional (função ventricular, presença/extensão de isquemia), com algumas evidências apontando superioridade da avaliação funcional sobre anatômica. ^4 - 6^

Para este propósito, sobretudo em pacientes com probabilidade pré-teste intermediária para DAC obstrutiva, a angiotomografia computadorizada de artérias coronárias (TCC) vem se destacando dentre os vários testes não invasivos como método robusto para descartar DAC obstrutiva, por seu elevado valor preditivo negativo. ^7^ Particularmente em estenoses moderadas (50% a 69%), a quantificação não invasiva da reserva fracionada de fluxo miocárdico (FFR _TC_ ) pode ajudar na correta discriminação de quais destas estão associadas à isquemia. ^8^ Estudos recentes demonstraram que a TCC tem elevada acurácia para identificar isquemia miocárdica por meio da quantificação não invasiva da FFRTC quando comparada ao padrão-ouro, a FFR invasiva pelo cateterismo cardíaco (FFRi). ^8 - 10^

A grande restrição do uso da FFR _TC_ na prática clínica têm sido sua baixa disponibilidade, especialmente devido à necessidade de *software* específico que requeria supercomputadores em grandes centros internacionais, encarecendo e prolongando substancialmente o processo. ^8^ Recentemente, um protótipo não comercial de *software* (disponível para computadores pessoais de configuração-padrão) que utiliza ferramentas de inteligência artificial – rede neural convolucional ( *deep learning* ) – para avaliação da FFR _TC_ foi testado por Rother et al., ^11^ Quando comparado com FFRi, a FFR _TC_ calculada por esse *software* demonstrou elevada acurácia para detecção de isquemia, com significativa redução no tempo de execução do seu cálculo quando comparado com modelos já existentes que utilizam supercomputadores. ^8^ Deve-se ressaltar, contudo, que este trabalho utilizou apenas imagens de um tomógrafo de última geração (Siemens Somatom Force – 384 cortes). Uma vez que esse *software* se propõe a calcular a FFR _TC_ em imagens adquiridas em tomógrafos com diferentes tecnologias, objetivamos utilizá-lo para investigar a acurácia diagnóstica da FFR _TC_ em tomógrafos de gerações anteriores, em comparação com FFRi, com qualidade de imagens que podem potencialmente afetar os resultados do algoritmo utilizado no *software* . Esse estudo também comparou a acurácia diagnóstica da FFR _TC_ com a avaliação anatômica isolada pela TCC.

## Métodos

### População do estudo

Retrospectivamente, este estudo incluiu pacientes com sintomas sugestivos de DAC significativa encaminhados para TCC. Após os achados desse exame, foram encaminhados por decisão clínica para o cateterismo cardíaco (com intervalo menor de 30 dias) e que realizaram análise de FFRi no Hospital Sírio-Libanês (São Paulo-SP), entre janeiro de 2014 e fevereiro de 2018. No total, houve 17 exclusões: 14 por fatores limitantes para o cálculo da FFR _TC_ descriminados pelo fabricante da ferramenta (8 por lesão de tronco de coronária esquerda [TCE], óstios ou bifurcações; 6 pela presença de *stent* ); e 3 por imagem com qualidade insuficiente devido a calcificação excessiva e significativos artefatos de movimento. Durante o pós-processamento das imagens, não houve exclusão de pacientes por impossibilidade técnica de o *software* realizar a quantificação da FFR _TC._ Ressalta-se que as recomendações do fabricante com relação a lesões de TCE, óstios ou bifurcações têm sido seguidas também por outros autores, ^11^ e parecem estar relacionadas ao reconhecimento limitado das bordas anatômicas nesses cenários. Esse estudo foi aprovado pelo Comitê de Ética em Pesquisa do Hospital Sírio-Libanês.

### Aquisição de imagens da TCC

As imagens foram obtidas utilizando-se os tomógrafos Siemens Somatom Definition Flash de 256 cortes (resolução temporal – TR – de 75ms; resolução espacial – SR – de 0,30mm) e Somaton Definition AS+ de 128 cortes (TR de150ms; SR de 0,30mm) (Siemens Healthineers, Forchheim, Alemanha). O preparo do paciente seguiu recomendações das diretrizes atuais, incluindo jejum de 4h, punção venosa com jelco calibre 18G, preferencialmente à direita, e monitoramento eletrocardiográfico contínuo. ^12^ Sempre que necessário, para controle da frequência cardíaca (FC), foi administrado betabloqueador (tartarato de metoprolol 50 a 100mg oral 1h antes do exame e/ou 5 a 20mg endovenoso poucos minutos antes da aquisição), objetivando-se manter a FC em torno de 55 a 60bpm. Todos os pacientes receberam ainda nitrato sublingual (isordil 2,5mg) poucos minutos antes da aquisição, exceto nos casos de hipotensão sintomática ou uso de inibidores de fosfodiesterase tipo 5 (de acordo com o tempo de ação de cada fármaco).

A aquisição das imagens foi planejada após a realização de teste *bolus* para cálculo do tempo de pico do contraste na aorta, com volume de 10 a 15mL, seguido de 30 a 50mL de soro fisiológico a 4,5 a 5,5mL/s. As imagens foram adquiridas utilizando o modo *Flash* (no tomógrafo *Definition Flash* ) ou de forma retrospectiva (em ambos os tomógrafos) com acoplamento eletrocardiográfico na diástole (55% a 75% do RR), corrente do tubo de 100 a 120kVp (ajustado pelo índice de massa corporal [IMC] individual), tempo de rotação de 0,28 ( *Flash* )/0,33 (AS+) segundos, 160 a 320mAs e espessura de corte de 0,6/0,3mm. A infusão de contraste iodado Optiray 350 (Ioversol 350mg/mL, Mallinckrodt-EUA) utilizou os mesmos parâmetros do teste *bolus* (60 a 90mL).

### Análise de imagens da TCC e FFRct

A análise das imagens da TCC foi realizada por meio do *software* Syngo.via ( *Siemens Healthineers, Forchheim, Germany* ). Após a escolha de imagens de melhor qualidade técnica, foram feitas avaliações da árvore coronariana em reformatação tridimensional, multiplanar e curva ( *vessel probe* ), com quantificação do grau de estenose e composição predominante da placa, quando presente (não calcificada, calcificada e mista). A quantificação da redução luminal foi realizada conforme recomendação da Sociedade de Tomografia Computadorizada Cardiovascular: ^13^ ausente, mínima (<25%), discreta (25% a 49%), moderada (50% a 69%), importante (70% a 99%) e oclusão (100%). As estenoses foram também classificadas como obstrutivas (≥50%) e não obstrutivas (<50%) pela TCC.

O pós-processamento da FFR _TC_ foi feito na mesma série em que foi realizada a análise visual anatômica (descrita anteriormente), utilizando a plataforma *Frontier* e o protótipo não comercial do *software* cFFR, versão 3.0 (Siemens Healthineers, Forchheim, Alemanha) por um médico experiente em imagem cardiovascular (>4 anos).

No cálculo da FFR _TC_ , inicialmente, foi realizada a detecção automática da linha central e dos contornos do lúmen das artérias coronárias, que foram revisados e corrigidos pelo médico especialista, quando necessário. Na sequência, o médico definiu o limite superior e inferior de todas as placas dos vasos que tiveram os valores de FFRi calculados no cateterismo.

Para a identificação correta do local em que se calculou FFRi, o médico hemodinamicista localizou o ponto de interesse nas imagens da fluoroscopia e documentou usando referências anatômicas (ramos das coronárias) e também um modelo de segmentação coronariana sugerido pela *Society of Cardiovascular Computed Tomography* (SCCT). O médico especialista em TCC utilizou essa documentação para selecionar a mesma localização anatômica das placas que tiverem a FFR _TC_ calculada.

**Figura 1:**
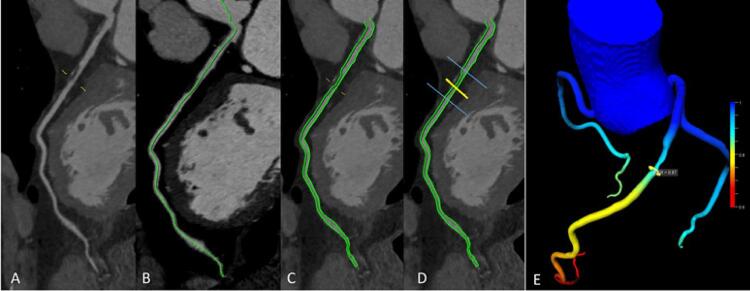
– Etapas para cálculo da FFR TC utilizando o software cFFR. A) Detecção visual da lesão coronariana. Definição do centerline B) e contornos luminais C) pelo software cFFR D) definição dos limites da lesão e do ponto de maior redução luminal pelo operador. E) Resultado do FFR no ponto de maior redução luminal demonstrado na árvore coronariana (após determinar centerline e contornos luminais nas três principais artérias coronárias: DA, Cx e CD).

O *software* cFFR calcula valores de FFR para toda a árvore coronariana com diâmetro ≥1,5mm e calcula automaticamente o valor ALM de cada placa delimitada. As etapas para cálculo da FFR _TC_ estão demonstradas na Figura 1 . Esse *software* foi desenvolvido com novas ferramentas de inteligência artificial, utilizando-se técnicas de aprendizado de máquina ( *deep learning* ). Todos eles foram instalados em computador padrão para laudos de radiologia. O tempo total de pós-processamento de todas as etapas foi em torno de 10 minutos.

### Cateterismo cardíaco e análise de FFRi

O cateterismo cardíaco foi realizado via radial ou femoral, utilizando cateteres diagnósticos de 6 ou 7 *french* (F). Para o cálculo de FFRi, o grau de estenose foi avaliado visualmente pelo hemodinamicista, utilizando-se pelo menos duas projeções ortogonais. As medidas de FFR foram realizadas com cateteres-guia de 6 ou 7F. Nitroglicerina intracoronária (0,2mg) foi injetada em todos os pacientes antes dos angiogramas. Um fio-guia de monitoramento de pressão foi posicionado distal à lesão-índice, e as pressões médias foram registradas no momento em que ficam estáveis. Adenosina intracoronariana foi injetada manualmente através do cateter-guia, em injeção de *bolus* de 80μg (artéria coronária esquerda) ou 40μg (coronária direita) em 10mL de soro fisiológico. Após sua administração, o menor valor de FFR estável durante o estado estacionário hiperêmico foi registrado. Esse valor corresponde à razão entre a pressão coronariana média distal à estenose e a pressão aórtica média no momento da hiperemia induzida farmacologicamente.

A exata posição do sensor de medida de FFRi foi documentada pelo hemodinamicista no laudo, e esta documentação foi utilizada pelo médico especialista em TCC para a medida da FFR _TC_ na mesma localização anatômica.

### Análise estatística

Análises descritivas foram expressas como frequência (porcentagem) para variáveis categóricas e como média ± desvio padrão para variáveis contínuas. A distribuição das variáveis contínuas foi avaliada por meio visual através de gráficos QQ e checada pelo teste de Shapiro-Wilk. Comparações entre as variáveis contínuas encontradas na TCC e no cateterismo (grau de estenose coronariana, FFR) dos pacientes foram realizadas pelo teste t de Student para amostras pareadas. Da mesma forma, correlação entre essas variáveis foi realizada por meio de correlação de Pearson.

Concordância entre FFR _TC_ e FFRi foi determinada por análise de Bland-Altman. Assumindo FFRi ≤0,8 como padrão-ouro para presença de isquemia, a *performance* diagnóstica da FFR _TC_ e de outros parâmetros anatômicos da TCC foi avaliada por meio do cálculo de sensibilidade, especificidade, valor preditivo positivo (VPP) e valor preditivo negativo (VPN). Além disso, a área sob a curva ROC (AUC) para detecção de lesões coronarianas associadas com isquemia foi calculada, e comparações entre as AUC foram realizadas de acordo com o método descrito por DeLong et al., ^14^ O melhor ponto de corte da FFR _TC_ para detecção de isquemia (FFRi ≤0,8) foi calculado utilizando-se o índice de Youden, que corresponde àquele com maior valor na equação (sensibilidade [Sens] + especificidade [Espe] – 1). ^15^ Ressalta-se que o grau de estenose foi explorado como variável contínua e categórica (DAC obstrutiva, >50%, ou não). A escolha pela forma categórica para inclusão no modelo se baseou em se tratar de um limiar clinicamente acionável para tomadas de decisões clínicas subsequentes.

Considerando a potencial correlação entre múltiplos vasos no mesmo indivíduo, o método de equações de estimação generalizadas com estrutura de correlação permutável foi usado para comparar amostras pareadas em um nível por-vaso. As análises estatísticas foram realizadas no *software* R ( *R Foundation for Statistical Computing* , Viena, Áustria). Todos os valores de p reportados são bicaudais e, quando <0,05, foram considerados estatisticamente significativos.

## Resultados

### Características dos pacientes e das placas

Noventa e três pacientes foram incluídos no estudo, com um total de 152 vasos. Cinquenta pacientes (54%) realizaram a TCC em tomógrafo Flash (256 colunas de detectores) e 43 (46%) em tomógrafo AS+ (128 colunas de detectores). A FC média nas aquisições foi de 58 ± 8bpm.

Setenta e quatro pacientes (80%) apresentavam DAC obstrutiva (estenose >50%) na TCC, sendo 48 com estenose moderada (50% a 69%) e 26 importante (>70%). Na análise por-vaso, as placas foram mais frequentemente mistas (70%), com localização mais comum (73%) na coronária descendente anterior (DA) e com ALM média de 3,2 ± 1,6mm ^2^ . Características clínicas e tomográficas dos pacientes estão expostas nas Tabelas 1 e 2 , respectivamente.

**Tabela 1:** – Dados demográficos

Variáveis	n = 93
Idade, anos*	64 ± 11
Sexo masculino, n (%)	70 (75)
Hipertensão, n (%)	54 (58)
Dislipidemia, n (%)	45 (48)
Diabetes, n (%)	24 (26)
Tabagismo, n (%)	7 (8)
IMC, kg/m ^2^ *	28 ± 4
FC, bpm*	58 ± 8

IMC: índice de massa corporal; FC: frequência cardíaca. *média ± desvio padrão.

**Tabela 2:** – análise do FFRTC e FFRi

Por paciente	n = 93
Estenose ≥ 50%, n (%)	74 (80)
Estenose 50-69%, n (%)	48 (52)
Estenose ≥ 70%, n (%)	26 (28)
FFR _TC_ ≤ 0,8, n (%)	32 (34)
FFRi ≤ 0,8, n (%)	39 (42)
**Por vaso**	**n = 152**
**Localização**	
DA, n (%)	111 (73)
Cx, n (%)	26 (17)
CD, n (%)	16 (10)
Estenose ≥ 50%, n (%)	124 (82)
Estenose 50-69%, n (%)	95 (63)
Estenose ≥ 70%, n (%)	29 (19)
ALM, mm ^2^ *	3,2 ± 1,6
**Morfologia**	
Calcificada, n (%)	16 (10)
Mista, n (%)	106 (70)
Não calcificada, n (%)	30 (20)
FFR _TC_ *	**0,88 ± 0,08**
FFR _TC_ ≤ 0,8, n (%)	32 (21)
FFRi*	**0,86 ± 0,08**
FFRi ≤ 0,8, n (%)	47 (31)

DAC: doença arterial coronariana; FFR: reserva fracionada de fluxo invasiva; FFR _TC_: reserva fracionada de fluxo por tomografia; ALM: área luminal mínima. ^*^ média ± desvio padrão.

### Comparação de FFR TC com FFRi

**Figura 2:**
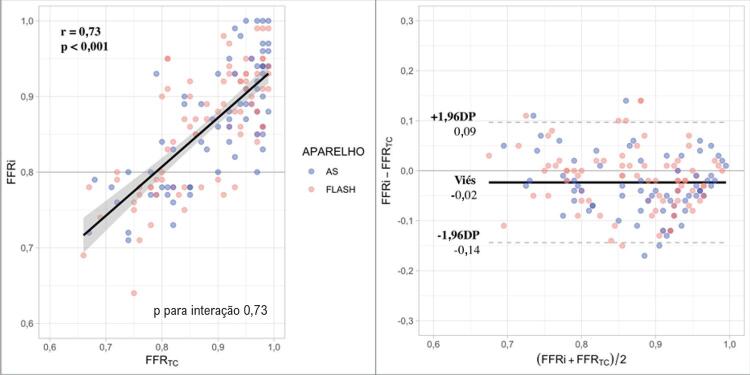
– Correlação (A) e concordância por análise de Bland-Altman (B) entre FFR TC e FFRi (análise por vaso): APARELHO: AS refere-se a tomógrafo de 128 cortes e FLASH, a tomógrafo de 256 cortes.

Houve forte correlação entre os valores da FFR _TC_ e FFRi (r = 0,73, p<0,001) ( Figura 2 ). Na média, os valores da FFR _TC_ foram discretamente superiores aos encontrados em FFRi (0,88 ± 0,08 *vs.* 0,86 ± 0,08, p = 0,02), erro sistemático que se confirma na análise de Bland-Altman (viés de –0,02 com intervalo de confiança de –0,14 a 0,09) (ver Figura 2 ). O tipo de tomógrafo utilizado não mudou a relação entre FFRi e FFR _TC_ (p-valor para interação de 0,73).

### Detecção de isquemia

**Figura 3:**
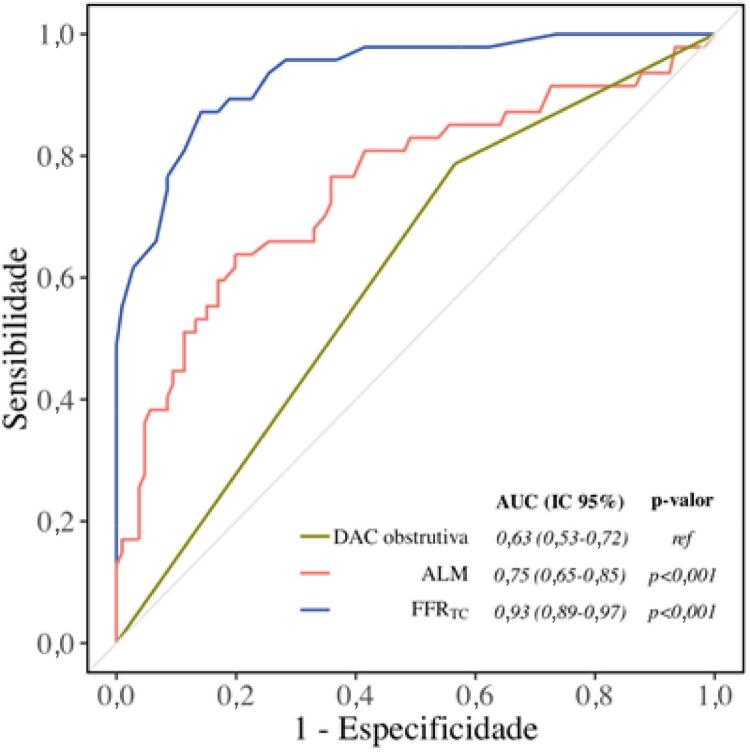
– Performance para o diagnóstico de lesão obstrutiva com limitação de fluxo (FFRi <0,8).

**Figura 4:**
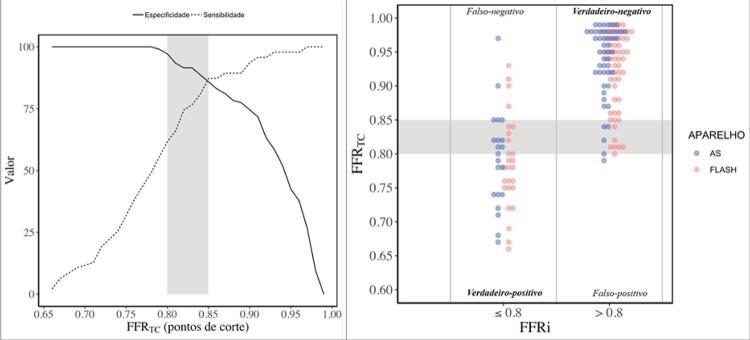
– Performance diagnóstica do valor de FFR TC <0,85.APARELHO: AS refere-se ao tomógrafo de 128 cortes e FLASH, ao tomógrafo de 256 cortes.

Para identificação de lesões coronarianas obstrutivas com limitação de fluxo (FFRi ≤0,8 como padrão-ouro), a FFR _TC_ demonstrou *performance* significativamente superior à classificação visual isolada de obstrução coronariana (AUC 0,93 *vs.* 0,61, p <0,001) e à ALM pela TCC (AUC 0,93 *vs.* 0,75, p <0,001) ( Figura 3 ). O melhor ponto de corte (com menor número de resultados falsos) para a FFR _TC_ definido por meio do índice de Youden, para discriminação de lesão com ou sem isquemia, foi 0,85, que apresentou valores de sensibilidade de 87%, especificidade de 86%, VPP de 73% e VPN de 94% nesse ponto de corte ( Figura 4 ). Tais métricas de *performance* utilizando-se esse ponto de corte (0,85) foram discretamente superiores analisando-se apenas as placas com redução luminal moderada (50% a 69%, n = 95), com sensibilidade de 89%, especificidade de 91%, VPP de 74%, VPN de 97%. Das 152 lesões avaliadas, 3 (2%) foram falso-positivas e 18 (12%), falso-negativas, utilizando-se o ponto de corte tradicional (FFR _TC_ ≤0,80). Por meio do ponto de corte mais elevado (FFR _TC_ < 0,85), 12 (7%) foram falso-positivas e 9 (6%), falso-negativas.

Ao avaliarmos o grau de redução luminal, as placas visualmente consideradas moderadas (50% a 69%) tiveram um número de falso-positivos de 86 (56%) em relação ao padrão-ouro (FFRi ≤0,8), enquanto as placas com redução luminal visual importante (≥70%) tiveram um número de falso-positivos de 23 (15%), o que, para esta última, representa uma magnitude 50% maior em relação aos resultados da FFR _TC_ <0,85 (15% *vs.* 7%).

## Discussão

A análise da FFR _TC_ através de *software* baseado em aprendizado de máquina demonstrou boa concordância em relação à medida de FFRi, destacando-se que o pós-processamento das imagens de TCC foi feito em computadores-padrão do próprio hospital e com tempo de pós-processamento em torno de 10min. Com relação à *performance* diagnóstica, mesmo em tomógrafos de gerações anteriores, a FFR _TC_ foi superior à avaliação anatômica isolada tanto na observação de estenoses coronarianas como no cálculo da ALM, reduzindo significativamente o número de falso-positivos.

Em linha com o demonstrado por Rother et al., ^11^ retrospectivamente em coorte com 71 pacientes e utilizando a mesma versão de *software* desse estudo (cFFR versão 3.0), ^11^ a FFR _TC_ apresentou considerável concordância com a medida de FFRi, com mínima superestimação. Esses resultados estão em desencontro com versões anteriores desse mesmo *software* (cFFR versão 1.4), ^16 - 19^ em que uma subestimação foi descrita, e provavelmente reflete mudanças do algoritmo com a atualização do *software* .

Apesar de comparável com os três principais estudos multicêntricos até hoje publicados (DISCOVER-FLOW, DeFacto and NXT), ^8 - 10^ há que se realçar que o limite de concordância do nosso estudo foi mais largo na análise de Bland-Altman (~0,20), que significa menor repetibilidade do método, em comparação com o observado por Rother et al., ^11^ Como, na média, os pacientes do nosso estudo apresentaram uma FC média <60 bpm, o que reflete uma boa qualidade das imagens em geral, acreditamos que o desempenho superior daquele estudo pode ser explicado, em parte, pela utilização de um tomógrafo com resolução espacial 20% superior (0,3 *vs.* 0,24mm), além do uso de um moderno algoritmo de reconstrução (ADMIRE). Esses fatores podem ter levado a uma melhor detecção dos contornos coronarianos (linha central e lúmen) por aquele estudo, com consequente melhora dos resultados. Outra justificativa que não pode ser descartada seria a maior experiência do observador daquele centro com a nova versão de cFFR.

Com relação ao poder de discriminação de estenoses coronarianas com/sem limitação de fluxo, a FFR _TC_ foi superior em comparação com a avaliação isolada anatômica da TCC, tanto qualitativamente (classificação visual de DAC obstrutiva) quanto quantitativamente (ALM). Utilizando-se um ponto de corte de 0,85 para a FFR _TC_ , os VPN e VPP foram comparáveis com os de outras coortes que utilizaram esse *software.* ^16 - 19^ Além disso, ressaltamos os seguintes aspectos: 1) a *performance* da FFR _TC_ foi melhor nos casos com lesões moderadas (50% a 69%); 2) a FFR _TC_ levou a uma redução de mais de 50% dos casos falso-positivos quando usada somente a avaliação anatômica de DAC grave (≥70%). Esses achados apresentam grande relevância na prática clínica, uma vez que lesões moderadas na TCC são relativamente frequentes e, muitas vezes, esses pacientes são encaminhados para exames adicionais. ^20^ De fato, a oportunidade de redução global de encaminhamento desnecessário para cateterismo pode ser ainda maior utilizando-se essa nova ferramenta de FFR _TC_ , uma vez que apenas 42% dos nossos pacientes apresentaram FFRi <0,8.

Por fim, destaca-se o rápido pós-processamento de imagens desse novo *software* com base em tecnologia de aprendizado de máquina ( *deep learning* ). Nos *softwares* pioneiros ^8 - 10^ que utilizam algoritmos de dinâmica de fluidos, o cálculo da FFR _TC_ leva de 1 a 4h de processamento, e é realizado em supercomputadores localizados apenas em centros específicos dos EUA (sede na Califórnia), Londres e Tóquio. Além do elevado custo, no geral, isso demanda cerca de 24h para a obtenção dos resultados e necessidade de enviar imagens DICOM para fora de ambientes da instituição. Portanto, esse novo *software* poderá promover impacto real na prática clínica no cuidado do paciente com DAC.

### Limitações

Trata-se de um estudo retrospectivo, unicêntrico, com uma população de estudo relativamente pequena e predominantemente apresentando DAC obstrutiva. Ao seguirmos as recomendações de aplicabilidade da ferramenta pelo fabricante, foram excluídos os pacientes com estenose significativa em tronco de coronária esquerda, óstios de coronárias principais ou em bifurcações; oclusões arteriais crônicas; história prévia de cirurgia de revascularização ou implante de *stent* . Da mesma forma, pacientes com sintomas típicos eventualmente não foram submetidos a exame funcional invasivo (FFRi) por decisão clínica. Portanto, esse estudo deve ser interpretado com a devida atenção ao contexto clínico da população participante (DAC menos grave/complexa e/ou cenários clínicos de maior dúvida diagnóstica).

## Conclusão

Essa nova versão da FFR _TC_ , mesmo em tomógrafos de gerações anteriores, apresentou boa *performance* diagnóstica na detecção de lesões coronarianas obstrutivas com limitação de fluxo, com redução expressiva do número de falso-positivos, o que pode reduzir significativamente a quantidade de pacientes que realizam testes adicionais. A importância clínica desses achados precisa ser validada por estudos especificamente desenhados para avaliações de desfechos clínicos. Esse *software* apresenta tecnologia inovadora, utilizando aprendizado de máquina, o que possibilita maior acessibilidade, rápida execução e, potencialmente, redução de custos.
