# Supplementary material for: Performance Diagnóstica da FFR por Angiotomografia de Coronárias através de Software Baseado em Inteligência Artificial
Source: Arq Bras Cardiol. 2021 Jun 8;116(6):1091–8. [Article in Portuguese] doi: 10.36660/abc.20190329 (PMC8288523; doi:10.36660/abc.20190329)
Supplement: Translation

# Translation: Diagnostic Performance of a Machine Learning-Based CT-Derived FFR in Detecting Flow-Limiting Stenosis

**Authors:** Thamara Carvalho Morais, Antonildes Nascimento Assunção-Jr, Roberto Nery Dantas, Carla Franco Grego da Silva, Caroline Bastida de Paula, Roberto Almeida Torres, Tiago Augusto Magalhães, César Higa Nomura, Luiz Francisco Rodrigues de Ávila, José Rodrigues Parga

**Affiliations:** 1 Brazil Hospital Sírio-libanês, São Paulo, SP - Brazil; 2 Brazil Universidade de São Paulo Faculdade de Medicina - CDI - InCor/HCFMUSP - Departamento de Imagem Cardiovascular, São Paulo, SP - Brazil; 3 Brazil Complexo Hospital de Clínicas da Universidade Federal do Paraná (CHC-UFPR) - Cardiovascular CT/MR, Curitiba, PR - Brazil

**Keywords:** Myocardial Fractional Flow Reserve, Coronary Artery Disease, Computed Tomography, Myocardial Ischemic, Machine Learning

## Abstract

**Background:**

The non-invasive quantification of the fractional flow reserve (FFRCT) using a more recent version of an artificial intelligence-based software and latest generation CT scanner (384 slices) may show high performance to detect coronary ischemia.

**Objectives:**

To evaluate the diagnostic performance of FFRCT for the detection of significant coronary artery disease (CAD) in contrast to invasive FFR (iFFR) using previous generation CT scanners (128 and 256- detector rows).

**Methods:**

Retrospective study with patients referred to coronary artery CT angiography (CTA) and catheterization (iFFR) procedures. Siemens Somatom Definition Flash (256-detector rows) and AS+ (128-detector rows) CT scanners were used to acquire the images. The FFRCT and the minimal lumen area (MLA) were evaluated using a dedicated software (cFFR version 3.0.0, Siemens Healthineers, Forchheim, Germany). Obstructive CAD was defined as CTA lumen reduction ≥ 50%, and flow-limiting stenosis as iFFR ≤0.8. All reported P values are two-tailed, and when <0.05, they were considered statistically significant.

**Results:**

Ninety-three consecutive patients (152 vessels) were included. There was good agreement between FFRCT and iFFR, with minimal FFRCT overestimation (bias: -0.02; limits of agreement:0.14-0.09). Different CT scanners did not modify the association between FFRCT and FFRi (p for interaction=0.73). The performance of FFRCT was significantly superior compared to the visual classification of coronary stenosis (AUC 0.93vs.0.61, p<0.001) and to MLA (AUC 0.93vs.0.75, p<0.001), reducing the number of false-positive cases. The optimal cut-off point for FFRCT using a Youden index was 0.85 (87% Sensitivity, 86% Specificity, 73% PPV, 94% NPV), with a reduction of false-positives.

**Conclusion:**

Machine learning-based FFRCT using previous generation CT scanners (128 and 256-detector rows) shows good diagnostic performance for the detection of CAD, and can be used to reduce the number of invasive procedures.

## Introduction

According to the most recent clinical guidelines,^1 - 3^ management of chronic and symptomatic coronary artery disease (CAD) may be guided by additional tests for either anatomical (extent, severity, morphology) or functional (ventricular function, presence/extent of ischemia) assessment, with evidence suggesting the superiority of the functional over the anatomical approach in some clinical scenarios.^4 - 6^

For this purpose, especially in patients with intermediate pretest probability of obstructive CAD, the coronary computed tomography angiography (CTA) stands out among the various existing non-invasive tests as a robust method to rule out obstructive CAD, given its high negative predictive value.^7^ Particularly in moderate stenosis (50-69%), the non-invasive measurement of myocardial fractional flow reserve (FFR_CT_) may help to correctly distinguish which of these are associated with ischemia^8^ . Recent studies have shown that the CTA is an accurate test to identify ischemia through non-invasive quantification of fractional flow reserve (FFR_CT_) when compared to the gold standard, the invasive FFR by cardiac catheterization (iFFR).^8 - 10^

The use of FFR_CT_in clinical practice has been mostly restricted by its low availability, especially due to the need for specific software that would run only on supercomputers at large international centers, substantially increasing the cost and delaying the whole diagnostic process.^8^ More recently, a non-commercial software prototype (available for standard configuration personal computers) that uses artificial intelligence tools – convolutional neural network (deep learning) – to evaluate FFR_CT_was tested by Rother et al.^11^ When compared to iFFR, the FFR_CT_calculated by this software has shown high accuracy to detect ischemia, with a significant reduction in the calculation time when compared to existing models that use supercomputers.^8^ However, it should be noted that their study used only images acquired by a latest generation CT scanner (Siemens Somatom Force – 384 slices). Since this software is capable of calculating the FFR_CT_from images acquired using CT scanners that employ different technologies, our aim was to use it to investigate the diagnostic accuracy of the FFR_CT_using previous generation CT scanners, compared to iFFR, with imaging quality that can potentially affect the results of the algorithm used in the software. This study also compared the diagnostic accuracy of FFR_CT_to the isolated anatomical assessment by CTA.

## Methods

### Study Population

Retrospectively, this study included symptomatic patients that were referred to CTA for investigating significant CAD and, after the test findings, upon clinical decision, were referred to cardiac catheterization (within less than 30 days) and underwent iFFR analysis at Sírio-Libanês Hospital, São Paulo, Brazil, between January 2014 and February 2018. There was a total of 17 exclusions: 14 owing to factors that limited the FFR_CT_calculation, as described by the tool manufacturer (8 due to left main coronary artery (LMCA), ostia, or bifurcation lesions, 6 owing to the presence of stent); and 3 due to bad quality image as a result of excessive calcification and significant motion artifacts. During image post-processing, no other patient was excluded as a result of the software technical inability to measure FFR_CT_. It is noteworthy that the manufacturer’s recommendations regarding LMCA, ostia, or bifurcation lesions have also been followed by other authors,^11^ and they seem to be related to the limited recognition of the anatomical borders in these scenarios. This study was approved by the Sírio-Libanês Hospital Research Ethics Committee.

### Acquisition of the CTA images

The images were obtained using a 256-slice Siemens Somatom Definition Flash CT scanner (temporal resolution - TR - of 75 ms; spatial resolution - SR - of 0.30 mm) and a 128-slice Somatom Definition AS CT scanner (TR of 150 ms; SR of 0.30mm) (Siemens Healthineers, Forchheim, Germany). The patients were prepared according to current guideline recommendations, including four-hour fasting, using an IV peripheral venous access catheter (18 Gauge), preferably on the right antecubital vein, and continuous electrocardiographic monitoring.^12^ Whenever necessary, a beta-blocker was administered (50-100mg oral metoprolol tartrate 1 hour before the test and/or 5-20mg intravenously a few minutes before image acquisition) to obtain heart rate (HR) control (aim 55-60 bpm). All patients also received sublingual nitrate (Isordil 2.5mg) a few minutes before acquisition, except in cases of symptomatic hypotension or use of phosphodiesterase type 5 inhibitors (according to each drug action onset).

Image acquisition was planned after bolus testing to calculate the contrast peak time in the aorta, with a 10-15 mL volume, followed by 30-50 mL of saline solution at 4.5-5.5 mL/s. The images were acquired using Flash mode (in the Definition Flash CT scanner) or retrospectively (in both CT scanners) with electrocardiographic gating in late diastole (55-75% of RR), tube voltage of 100-120 kVp (adjusted to the individual BMI), rotation time 0.28 (Flash) / 0.33 (AS+) seconds, 160-320 mAs and slice thickness of 0.6/0.3 mm. The Optiray 350 (Ioversol 350 mg/mL-Mallinckrodt-USA) iodinated contrast media infusion used the same parameters of the bolus testing (60-90ml).

### Analysis of the CTA and FFR CT images

CTA image analysis was carried out using the Syngo.via imaging software (Siemens Healthineers, Forchheim, Germany). After choosing the images with best technical quality, the coronary tree evaluation was performed by three-dimensional, curved, multiplanar reformation (Vessel Probe), with quantification of stenosis degree and prevalent plaque composition, when present (non-calcified, calcified, and mixed). Luminal reduction quantification was carried out according to the Society of Cardiovascular Computed Tomography recommendations:^13^ normal, minimal (<25%), mild (25-49%), moderate (50-69%), severe (70-99%) and occlusion (100%). CTA-assessed stenosis was also classified as obstructive (≥50%) and non-obstructive (<50%).

Post-processing FFR_CT_was performed in the same series in which visual anatomical analysis was carried out (as described above), using the Frontier platform and the non-commercial prototype of the cFFR software, version 3.0 (Siemens Healthineers, Forchheim, Germany), by a physician experienced in cardiovascular imaging (>4 years).

To calculate coronary artery FFR_CT_, the first step was to perform the automatic detection of the centerline and lumen contours, which were reviewed and corrected by the specialist, when necessary. Next, the upper and lower borders of all plaques in the vessels that had their iFFR values calculated during invasive catheterization were delimited.

To precisely identify the site where the iFFR was calculated, the interventional cardiologist located the point of interest in the fluoroscopic images and documented it using anatomical references (coronary branches) and also a coronary segmentation model suggested by the Society of Cardiovascular Computed Tomography (SCCT). The CTA expert used this documentation to select the same anatomical location of the plaques that had the FFR_CT_calculated.

The cFFR software calculates FFR values only for vessels ≥1.5mm in diameter and automatically calculates the value of the minimal lumen area (MLA) of each delimited plaque. The steps for FFR_CT_calculation are shown in Figure 1 . This software was developed with new artificial intelligence tools, using machine-learning techniques (deep learning). All of them were installed in a standard computer used for radiology reports. The total post-processing time of all steps was around 10 minutes.

**Figure 1:** – Steps to calculate FFRCT using the cFFR software. A) Visual detection of the coronary lesion. Definition of centerline B) and lumen contours C) using the cFFR software D) definition by the operator of the lesion borders and the point of higher lumen reduction. E) FFR result at the point with higher lumen reduction shown in the coronary tree (after determining the centerline and lumen contours in the 3 main coronary arteries: LAD, LCx, and RCA).

### Cardiac catheterization and iFFR analysis

Cardiac catheterization was performed through radial or femoral access, using 6 or 7 French (F) diagnostic catheters. To calculate the iFFR, the degree of stenosis was visually evaluated by the interventional cardiologist using at least two orthogonal projections. Intracoronary nitroglycerin (0.2 mg) was injected in all patients before the angiograms. A pressure monitoring guidewire was placed distal to the index lesion, and the mean pressures were recorded when they were stable. Intracoronary adenosine was manually injected through the guide catheter, through an 80 μg bolus injection (left coronary artery) or 40 μg bolus (right coronary artery) into 10 mL of saline solution. After the administration, the lowest stable FFR value during the hyperemic steady state was recorded. This value corresponds to the ratio between the mean coronary pressure distal to the stenosis and the mean aortic pressure at the time of the pharmacologically-induced hyperemia.

The exact position of the iFFR measurement sensor was documented by the interventional cardiologist on the report, and this documentation was used by the CTA expert to measure FFR_CT_at the same anatomical location.

### Statistical Analysis

Descriptive analyses were expressed as frequency (percentage) for categorical variables, and as mean ± standard deviation for continuous variables. The distribution of the continuous variables was visually assessed using QQ plots and checked using the Shapiro-Wilk test. Comparisons between the continuous variables found in the CTA and the catheterization (degree of coronary stenosis, FFR) were made using Student’s t-test for paired samples. Likewise, the correlation between these variables was made using Pearson’s correlation.

The agreement between FFR_CT_and iFFR was determined by Bland-Altman analysis. Assuming iFFR ≤0.8 as the gold standard for the presence of ischemia, the diagnostic performance of FFR_CT_and other CTA anatomical parameters were evaluated by calculating sensitivity, specificity, positive predictive value (PPV) and negative predictive value (NPV). In addition, the area under the ROC curve (AUC) to detect coronary lesions associated with ischemia was calculated, and comparisons between the AUCs were performed according to the method described by DeLong et al.^14^ The best FFR_CT_cut-off point for the detection of ischemia (iFFR ≤0.8) was calculated using the Youden index, which corresponds to the one with the highest value in the equation (sensitivity [Sens] + specificity [Spec] - 1).^15^ It is noteworthy that the degree of stenosis was explored as a continuous and categorical variable (obstructive CAD, >50%, or not). The choice to include the categorical form in the model was based on the fact that it is a clinical threshold for further decision-making.

Considering the potential correlation between multiple vessels in the same individual, the generalized estimating equations (GEE) method with exchangeable correlation structure was used at a per-vessel level. Statistical analyses were carried out using the R software (R Foundation for Statistical Computing, Vienna, Austria). All reported *P* values are two-tailed, and when <0.05, they were considered statistically significant.

## Results

### Characteristics of the patients and plaques

Ninety-three patients were included in the study, with a total of 152 vessels. Fifty patients (54%) underwent CTA in the Flash CT scanner (256-detector rows), and 43 (46%) in the AS+ CT scanner (128-detector rows). The average HR during image acquisition was 58 ± 8 bpm.

Seventy-four patients (80%) showed obstructive CAD (stenosis >50%) in the CTA, 48 with moderate stenosis (50-69%) and 26 with severe stenosis (>70%). In the per-vessel analysis, plaques were more often the mixed type (70%), and most commonly located in the left anterior descending coronary artery (LAD) (73%) and had a mean MLA of 3.2 ± 1.6 mm^2^. Clinical and CT characteristics of the patients are shown in Tables 1 and 2 , respectively.

**Table 1:** – Demographic Data

Variables	n = 93
Age, years*	64 ± 11
Male, n (%)	70 (75)
Hypertension, n (%)	54 (58)
Dyslipidemia, n (%)	45 (48)
Diabetes, n (%)	24 (26)
Smoking, n (%)	7 (8)
BMI, kg/m^2^*	28 ± 4
HR, bpm*	58 ± 8

BMI: body mass index; HR: heart rate. *mean ± standard deviation.

**Table 2:** – Coronary plaque characterization

Per patient	n = 93
Stenosis ≥ 50%, n (%)	74 (80)
Stenosis 50-69%, n (%)	48 (52)
Stenosis ≥ 70%, n (%)	26 (28)
FFR_CT_≤ 0.8, n (%)	32 (34)
iFFR ≤ 0.8, n (%)	39 (42)
**Per vessel**	**n = 152**
Location	
LAD, n (%)	111 (73)
LCx, n (%)	26 (17)
RCA, n (%)	16 (10)
Stenosis ≥ 50%, n (%)	124 (82)
Stenosis 50-69%, n (%)	95 (63)
Stenosis ≥ 70%, n (%)	29 (19)
MLA, mm^2^*	3.2 ± 1.6
**Morphology**	
Calcified, n (%)	16 (10)
Mixed, n (%)	106 (70)
Non-calcified, n (%)	30 (20)
FFR_CT_*	**0.88 ± 0.08**
FFR_CT_≤ 0.8, n (%)	32 (21)
iFFR*	**0.86 ± 0.08**
iFFR ≤ 0.8, n (%)	47 (31)

CAD: coronary artery disease; iFFR: invasive fractional flow reserve; FFR_CT_: fractional flow reserve derived from computed tomography; MLA: minimal lumen area. *mean ± standard deviation.

### Comparison between FFR CT and iFFR

There was a strong correlation between the FFR_CT_and iFFR values (r = 0.73, p<0.001) ( Figure 2 ). On average, FFR_CT_values were slightly higher than iFFR values (0.88 ± 0.08 vs. 0.86 ± 0.08, p = 0.02), a systematic error confirmed by the Bland-Altman analysis (bias of -0.02 with a confidence interval of -0.14 to 0.09) ( Figure 2 ). The type of CT scanner used did not change the association between iFFR and FFR_CT_(p-value for interaction of 0.73).

**Figure 2:** – Correlation (A) and agreement using Bland-Altman analysis (B) between FFR CT and iFFR (per-vessel analysis): EQUIPMENT: AS refers to the 128-detector row CT scanner and FLASH to the 256-detector row CT scanner.

### Ischemia Detection

For the identification of flow-limiting obstructive coronary lesions (iFFR ≤0.8 as the gold standard), FFR_CT_showed a significantly superior performance compared to the isolated visual classification of coronary obstruction (AUC 0.93 vs. 0.61, p<0.001) and to MLA by CTA (AUC 0.93 vs. 0.75, p<0.001) ( Figure 3 ). The best cut-off point (with fewer false-positive results) for FFR_CT_, defined using the Youden index, to distinguish lesions with from those without ischemia was 0.85, which achieved the following accuracy values: 87% sensitivity, 86% specificity, 73% PPV, and 94% NPV at this cut-off point ( Figure 4 ). These performance metrics using this cut-off point (0.85) were slightly higher when analyzing only the plaques with moderate lumen reduction (50-69%, n=95), with 89% sensitivity, 91% specificity, 74% PPV, 97% NPV. Out of the 152 evaluated lesions, three (2%) were false-positives and 18 (12%) were false-negatives using the traditional cut-off point (FFR_CT_≤ 0.80). Using the highest cut-off point (FFR_CT_<0.85), 12 (7%) were false-positives and 9 (6%) false-negatives.

**Figure 3:** – FFR CT performance for the diagnosis of flow-limiting obstructive lesions (iFFR<0.8).

**Figure 4:** – Diagnostic performance of FFR CT <0.85. EQUIPMENT: AS refers to the 128-detector row CT scanner and FLASH to the 256-detecttor row CT scanner.

When evaluating the lumen reduction degree, the plaques with visually moderate reduction (50-69%) showed 86 (56%) false-positive cases in relation to the gold standard (iFFR ≤0.8), while the plaques with visually severe lumen reduction (≥70%) showed 23 (15%) false-positive cases, which for the latter represent a 50% higher magnitude when compared to the results of FFR_CT_<0.85 (15% *versus* 7%).

## Discussion

FFR_CT_analysis using a machine learning-based software showed good agreement with iFFR measurements, emphasizing that CTA image post-processing was carried out using standard computers in about 10 minutes. Regarding its diagnostic performance, even using previous-generation CT scanners, FFR_CT_performed better than the isolated anatomical evaluation, both in visual stenosis quantification and in the calculation of MLA, significantly reducing the number of false-positives.

In agreement with Rother et al.,^11^ which retrospectively studied a cohort of 71 patients using the same software version used in this study (cFFR version 3.0),^11^ the FFR_CT_showed considerable agreement with the iFFR measurement, with minimal overestimation. These results are in disagreement with previous versions of this same software (cFFR version 1.4),^16 - 19^ where an underestimation was described, which probably reflects algorithm changes with the software upgrade.

Although comparable to the three major multicenter studies published to date (DISCOVER-FLOW, Defacto, and NXT),^8 - 10^ it should be noted that the limit of agreement of our study was wider in the Bland-Altman analysis (~0.20), which means lower repeatability of the method, compared to what was observed by Rother et al.^11^ As our patients had an HR <60 bpm on average, which results in good image quality, we believe that the superior performance of that study can be explained, in part, by the use of a CT scanner with 20% higher spatial resolution (0.3 vs. 0.24mm), in addition to the use of a more recent and robust reconstruction algorithm (ADMIRE). These factors may have led to a better detection of coronary contours (centerline and lumen) by that study, with consequent better results. Another justification that cannot be ruled out would be the broader experience by the observer with the new version of cFFR at that center.

Regarding the power to distinguish flow-limiting from non-flow-limiting coronary stenosis, the FFR_CT_was superior in comparison to the CTA isolated anatomical evaluation, both qualitatively (visual classification of obstructive CAD), and quantitatively (MLA). Using a cut-off point of 0.85 for the FFR_CT_, the NPV and PPV were comparable to those of other cohorts that used this software.^16 - 19^ In addition, we point out the following aspects: 1) FFR_CT_performance was better in cases with moderate lesions (50-69%); 2) FFR_CT_led to a reduction of over 50% in the number of false-positive cases observed when only the anatomical evaluation of severe CAD (≥70%) was used. These findings are highly relevant in clinical practice, since moderate lesions in CTA are relatively frequent and often these patients are referred to additional tests.^20^ In fact, the opportunity of global reduction in unnecessary referrals to catheterization may be even greater using this new FFR_CT_tool, since only 42% of our patients had iFFR <0.8.

Finally, we point out this new software fast image post-processing based on machine-learning technology (deep learning). When using pioneering softwares,^8 - 10^ which use computational fluid dynamic algorithms, the calculation of the FFR_CT_takes from 1 to 4 hours to be processed, and it is carried out by supercomputers located only in specific centers in the United States (whose headquarters is in California), the UK, and Japan. In addition to the high cost, in general, about 24 hours are required to obtain the results, and the DICOM images need to be sent out of the institution environment. Therefore, this new software could have a real impact on clinical practice for the care of patients with CAD.

### Limitations

This is a retrospective, unicentric study, with a relatively small study population, which predominantly had obstructive CAD. When following the manufacturer’s recommendations regarding the tool appropriate use, patients with significant stenosis in the left main coronary artery, main coronary ostia, or in bifurcations; chronic arterial occlusions; previous history of revascularization surgery or stent implantation were excluded. Likewise, patients with typical symptoms were not always submitted to invasive functional testing (iFFR) by clinical decision. Therefore, this study should be interpreted while giving due attention to the clinical context of its population (less severe/complex CAD and/or clinical scenarios of greater diagnostic uncertainty).

## Conclusion

This new version of FFR_CT_, even when using previous-generation CT scanners, showed good diagnostic performance for the detection of flow-limiting obstructive coronary lesions, with a significant reduction in the number of false-positive cases, which can significantly decrease the number of patients who are referred to additional tests. The clinical importance of these findings needs to be validated by studies specifically designed to evaluate clinical outcomes. This software features innovative technology that uses machine learning, which enables greater accessibility, rapid performance and potential cost reduction.
